# Protective efficacy of natansnin, a dibenzoyl glycoside from *Salvinia natans *against CCl_4 _induced oxidative stress and cellular degeneration in rat liver

**DOI:** 10.1186/1471-2210-10-13

**Published:** 2010-10-12

**Authors:** Polimetla Srilaxmi, Gangadhara Reddy Sareddy, Polavarapu Bilhan Kavi kishor, Oruganti Hussainaiah Setty, Phanithi Prakash Babu

**Affiliations:** 1 Department of Genetics, Osmania University, Hyderabad, India; 2 Department of Biochemistry, University of Hyderabad, Hyderabad, India; 3 Department of Biotechnology, University of Hyderabad, Hyderabad, India

## Abstract

**Background:**

Carbon tetra chloride (CCl_4_), an industrial solvent, is a hepatotoxic agent and it is the well established animal model for free radical-induced liver injury. The present investigation was carried out to establish the protective effect of natansnin, a novel dibenzoyl glycoside from *Salvinia natans *against CCl_4 _induced oxidative stress and cellular degeneration in rat liver.

**Results:**

CCl_4 _significantly increased the levels of lipid peroxides, oxidized glutathione and decreased the levels of reduced glutathione, SOD and CAT. CCl_4 _induce marked histopathological changes and increase in the levels of apoptotic proteins. CCl_4 _treatment significantly increased the levels of apoptotic proteins such as caspases-3, PARP, Bax, Bid and cytochrome C and also increased the levels of inflammatory mediators iNos and Cox-2. Natansnin treatment significantly decreased the levels of CCl_4 _induced apoptotic proteins and inflammatory mediators. Further natansinin treatment significantly inhibited the CCl_4 _induced apoptosis which was evident form the reduced TUNEL positive cells.

**Conclusions:**

In conclusion, our study demonstrated the protective effect of natansnin against CCl_4 _induced oxidative stress and cellular degeneration in rat liver tissue. This protective effect of natansnin can be correlated to its direct antioxidant effect.

## Background

According to World Health Organization (WHO) more than 80% of the world's populations in developing countries depend primarily on herbal medicine for basic healthcare needs [1]. ***Salvinia natans ***is a free floating, rootless aquatic fern and belongs to the family Salvinaceae. Ferns of the genus Salvinia comprise of ten species; majority of which grow in freshwater bodies of tropical and subtropical regions, mainly in Africa and South America [2]. *Salvinia *varieties have small hairs on their leaves, making them water resistant. Air cavities in the leaves help the tiny plant to stay afloat. For many years, aquatic plants were used as an effective strategy for decontaminating waste water. *Salvinia natans *was found to have a great potentiality for the removal of heavy metals like lead (Pb), cadmium (Cd), nickel (Ni), copper (Cu), chromium (Cr) and mercury (Hg) from waste water. The understanding of chemistry of Salvinia plants can help controlling their invasive growth, and promote their utilization for useful purposes [3]. The phytochemical investigation on *S. natans *showed that it consists of 96% of amino compounds [4]. Though it is used in Homeopathy and other systems of medicine, no reports on medicinally important phytochemical compounds isolated from Salvinia natans. Several reports indicate that there is an inverse relationship between the dietary intake of antioxidant-rich foods and the incidence of human diseases [5,6]. Hence search for new synthetic and natural antioxidants is essentially important. For the present study, the plants were collected from the Chidambaram area of TamilNadu, India. Plants were collected in the month of November, 2005. Prof. R. Pannerselvam, Department of Botany, Annamalai University, Annamalai Nagar 608 002, TamilNadu, India, identified the plant. Herbarium of the Department of Botany, Annamalai Nagar 608 002, Tamil Nadu, India. Herbarium sheet number is 670. In the present study, natansnin, a dibenzoyl glycoside was isolated from *S.natans *and found to have antioxidative properties.

CCl_4 _is a common industrial solvent which is well-known for its hepatotoxicity [7-9]. CCl_4 _is commonly used for free radical induced liver injury. Liver is not the only target organ of CCl_4 _but it also effect several organs like kidneys, lungs, heart, testis, brain and blood [10-12]. Through the investigation of acute CCl_4 _induced liver damage in animal models, it is now generally accepted that CCl_4 _toxicity results from bioactivation of CCl_4 _into trichloromethyl free radical by cytochrome P450 system in liver microsomes and consequently causes lipid peroxidation of membranes that leads to liver injury [13-15]. Lipid peroxidation initiated by free radicals is considerably deleterious for cell membranes and implicated in a number of pathological conditions. In the present study we examined the protective efficacy of natansnin isolated from *Salvinia natans *against CCl_4 _induced oxidative stress and cellular degeneration in rat liver.

## Results

### Isolation of compounds from *Salvinia natans*

Secondary plant products are isolated from *Salvinia natans *using column chromatography. A total of five compounds (fatty acid, simple benzaldehyde, triterpenoid and two glycosides) were obtained from whole plants of *Salvinia natans *and their chemical structures characterized. One of the glycoside was identified as an unusual, novel, 1, 2- dibenzoyl glycoside after structural elucidation. The molecular formula was determined as C_20 _H_20 _O_13 _with a molecular weight of 468. The compound is named as natansnin. Isolation, purification and structural elucidation of natansnin accepted in journal of Natural products research. The article is in the press. (An unusual novel anti-oxidant dibenzoyl glycoside from *Salvinia natans *by M. Narasimhulu, K. Ashalatha P. Sri Laxmi, A. V. S. Sarma, B. Rama Rao, P.B. Kavi Kishor, G. L. David Krupadanam, A. Zehra Ali, Asok K. Tiwari, A. Panneer Selvam and Y. Venkateswarlu).

Natansnin was screened for antioxidant activity using 2, 2'-di phenyl-1-picrylhydrazyl (DPPH) radical scavenging method in *in vitro *is shown in Table 1. A known antioxidant butylated hydroxy toluene (BHT) was used as standard. The antioxidant activity of natansnin compared with known antioxidant BHT. The activity expressed as % inhibition of radical scavenging activity. While natansnin showed 60.6% of radical scavenging activity, BHT showed 63.6%.

**Table 1:** Effect of natansnin on DPPH* radical scavenging activity

Compound	% inhibition of radical scavenging activity
Butylated hydroxy toluene (BHT)	60.6

Natansnin	63.6

Natansnin was screened for antioxidant activity using 2, 2'-di phenyl-1-picrylhydrazyl (DPPH) radical scavenging method. A known antioxidant butylated hydroxy toluene (BHT) was used as standard. The activity expressed as % inhibition of radical scavenging activity. While natansnin showed 60.6% of radical scavenging activity, BHT showed 63.6%.

The effect of administration of CCl_4 _on oxidative stress and cellular degeneration and the protective effect of natansnin on CCl_4 _induced toxicity were studied. Results of all the parameters in this study were expressed relative to control, which was taken as 100. The actual values for control group are given in the corresponding figures. In other words a 100% protection (on a given parameter) means that the value is back to the control level which is normalized as 100.

### Natansnin reduced the levels of serum enzymes induced by CCl_4_

A significant increase in the activity of the serum enzymes ALP and ALT were observed in rats receiving CCl_4 _in vehicle (Group 2) when compared to normal (Group 1) rats administered vehicle alone is shown in Table 2. However, the activities of these serum enzymes were significantly (p < 0.05) lower in rats treated with natansnin (Group 4 and 5) than in Group 2 rats.

**Table 2:** Effect of natansnin on levels of serum enzymes induced by CCl_4_

	ALT (IU/L)	ALP(IU/L)
Control	100	100

CCl_4_	238.26 ± 22.2^a^	244.82 ± 18.6^a^

Natansnin (20 mg/kg body wt)	108.26 ± 11.7	114.13 ± 10.9

Natansnin 10 mg + CCl_4_	173.45 ± 16.2^a, b^	162.27 ± 14.5^a, b^

Natansnin 20 mg + CCl_4_	165.61 ± 15.3^a, b, c^	145.61 ± 13.6^a, b, c^

Effect of CCl_4 _with or without prior administration of natansnin on levels of serum enzymes in liver. Values are expressed as percent response compared to control rats. The control values of ALT and ALP were 53.43 ± 3.6, 285.28 ± 18.2 IU/L respectively. a = Statistical significant at *P < 0.05 *as compare to control, b = Statistical significant at *P < 0.05 *as compare to CCl_4_, c = Statistical significant at *P < 0.05 *as compare to natansnin (10 mg) + CCl_4_.

### Natansnin reduced lipid peroxidation induced by CCl_4_

**Figure 1:**
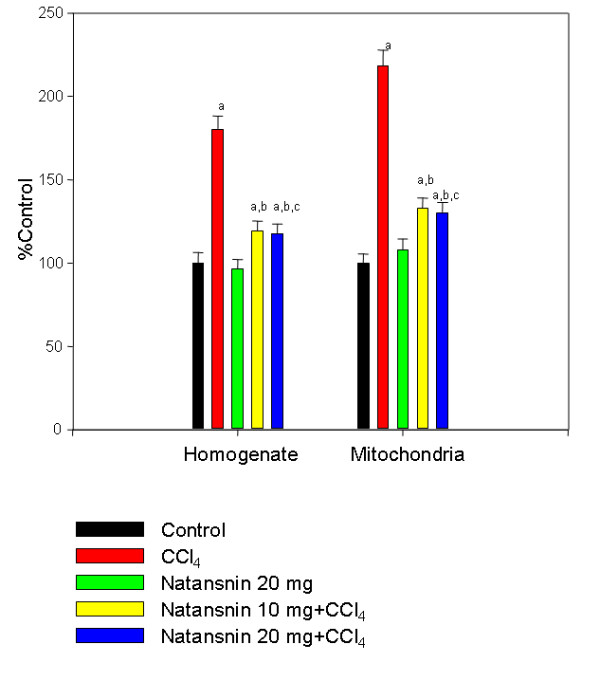
**Effect of CCl_4 _with or without prior administration of natansnin on lipid peroxide level in liver homogenate and mitochondria**. Homogenate and mitochondrial fractions were isolated from control, CCl_4 _and natansnin (10 mg/kg, 20 mg/kg body weight) treated rats and lipid peroxides were estimated using thiobarbituric acid reaction method. Values are given as percent control, and are mean ± S.D. of at least four animals. Lipid peroxide levels were expressed as nmol MDA formed per 100 mg protein. The control values in homogenate and mitochondria are 135.2 ± 8.6, 121.4 ± 6.6 respectively. a = Statistical significant at *P < 0.05 *as compare to control, b = Statistical significant at *P < 0.05 *as compare to CCl_4_, c = Statistical significant at *P < 0.05 *as compare to CCl_4_+ natansnin (10 mg).

Effect of the administration of CCl_4 _with and without the prior administration of natansnin on lipid peroxide level is shown in Figure 1. In homogenate and mitochondria, the lipid peroxide levels significantly increased (80 and 118% respectively) due to the administration of CCl_4 _compared to controls. (Control = 135.2 ± 8.6 nmol/100 mg protein in homogenate and 121.4 ± 6.6 nmol/100 mg protein in mitochondria) (CCl_4 _= 244.3 ± 10.1 nmol/100 mg protein in homogenate and 265.3 ± 11.2 nmol/100 mg protein). There was a significant decrease in the level of lipid peroxides in both homogenate and mitochondria, in natansnin and CCl_4 _treated rats. Prior administration of 10 mg/kg body wt of natansnin offered a protection rate of 61 and 86% in homogenate (161.5 ± 7.8 nmol/100 mg protein) and mitochondria (161.4 ± 7.5 nml/100 mg protein). With an increase of natansnin to 20 mg/kg body wt, protection rate increased only marginally 63 and 88% in homogenate (159.4 ± 7.5 nmol/100 mg protein) and (158.6 ± 7.2 nmol/100 mg protein) mitochondria respectively. Administration of natansnin alone did not show any change on formation of lipid peroxides when compared to control animals.

### Natansnin restored GSH/GSSG ratio

**Figure 2:**
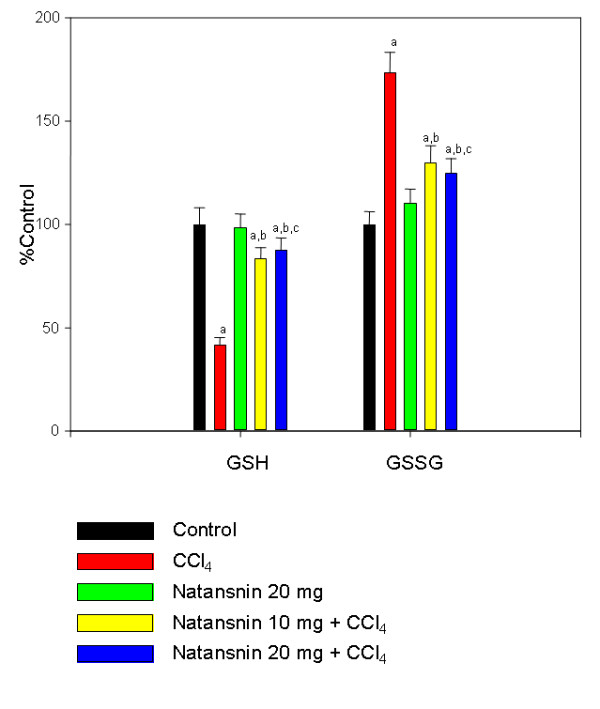
**Effect of CCl_4 _with or without prior administration of natansnin on glutathione (oxidized & reduced) levels in liver**. Reduced and oxidized glutathione levels were measured in liver homogenate from control, CCl_4 _and natansnin (10 mg/kg, 20 mg/kg body weight) treated rats. Values are given as percent control, and are mean ± S.D. of at least four animals. Glutathione levels are expressed as μmoles per gram tissue. The control values of GSH and GSSG were 32.12 ± 2.6, 11.3 ± 0.71 respectively. a = Statistical significant at *P < 0.05 *as compare to control, b = Statistical significant at *P < 0.05 *as compare to CCl_4_, c = Statistical significant at *P < 0.05 *as compare to CCl_4_+ natansnin (10 mg).

Effect of CCl_4 _with and without the prior administration of natansnin on levels of glutathione (both oxidized and reduced) is shown in Figure 2. The levels of reduced glutathione (GSH) were decreased by 48.1% in CCl_4 _treated rats, when compared to controls. Natansnin treatment at 10 mg/kg body weight gave a protection rate of 41.59% and at 20 mg/kg body wt, it slightly improved it (45%). The levels of oxidized glutathione (GSSG) increased by 73% in CCl_4 _treated rats when compared to controls. Natansnin treatment at 10 mg/kg body wt protected the rats by 43% and at 20 mg/kg body wt up to 48%. There was a significant difference in rats administered both natansnin and CCl_4 _(group 4 and 5) when compared to rats administered with only CCL_4_. There was no significant effect on glutathione levels in rats administered with natansnin alone (group 3). The ratio of GSH/GSSG is shown in Table 3.

**Table 3:** GSH/GSSG Ratio

	GSH (μ mol/gm tissue)	GSSG (μ mol/gm tissue)	Ratio
Control	32.12 ± 2.6	11.3 ± 0.71	0.84
CCl_4 _treated	16.7 ± 1.05	19.6 ± 1.11	-1.14
Natansnin treated	30.03 ± 2.4	11.5 ± 0.85	0.65
Natansnin (10 mg) + CCl_4 _treated	26.8 ± 1.7	14.7 ± 0.9	-0.16
Natansnin (20 mg) + CCl_4 _treated	28.1 ± 1.91	14.1 ± 0.81	-0.007

Reduced and oxidized glutathione levels were measured in liver homogenate from control, CCl_4 _and natansnin (10 mg/kg, 20 mg/kg body weight) treated rats. Glutathione levels are expressed as μmoles per gram tissue. Ratio = GSH-2(GSSG)/GSSG.

### Natansnin restored activities of antioxidant enzymes: catalase and superoxide dismutase

**Figure 3:**
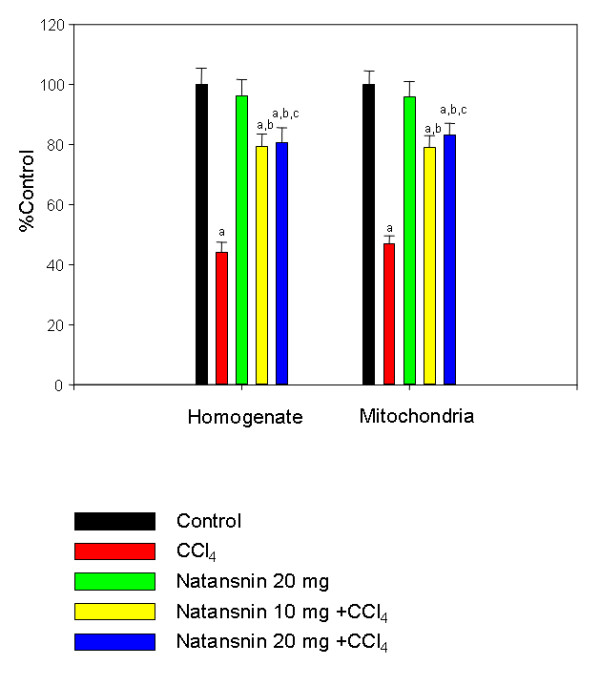
**Effect of CCl_4 _with or without prior administration of natansnin on Catalase levels in liver homogenate and mitochondria**. Catalase levels were estimated in homogenate and mitochondrial fractions from control, CCl_4 _and natansnin (10 mg/kg, 20 mg/kg body weight) treated rats. Values are given as percent control, and are mean ± S.D. of at least four animals. 0.3 mg of homogenate protein and 0.1 mg of mitochondrial protein were used for each assay. Catalase activity is expressed as nmols of H_2_O_2 _consumed per min per mg protein. The control values in homogenate and mitochondria are 733.9 ± 38.9, 1453 ± 64.6 respectively. a = Statistical significant at *P < 0.05 *as compare to control, b = Statistical significant at *P < 0.05 *as compare to CCl_4_, c = Statistical significant at *P < 0.05 *as compare to CCl_4_+ natansnin (10 mg).

**Figure 4:**
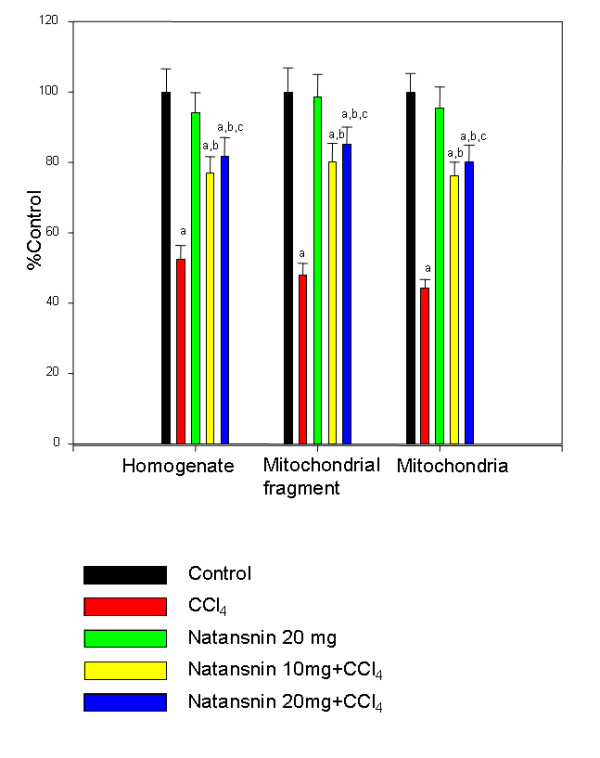
**Effect of CCl_4 _with or without prior administration of natansnin on SOD levels in liver homogenate and mitochondria**. Superoxide dismutase levels were estimated in homogenate, mitochondrial fragment and pure mitochondria from control, CCl_4 _and natansnin (10 mg/kg, 20 mg/kg body weight) treated rats. Values are given as percent control, and are mean ± S.D. of at least four animals. 0.3 mg of homogenate protein and 0.1 mg protein of sample (mitochondrial fragment and mitochondria) were used for each assay. Super oxide dismutase activity is expressed as units per mg protein. The control values of super oxide dismutase of homogenate and mitochondrial fragment and intact mitochondria were 2.88 ± 0.19, 3.08 ± 0.21 and 4.73 ± 0.25 respectively. a = Statistical significant at *P < 0.05 *as compare to control, b = Statistical significant at *P < 0.05 *as compare to CCl_4_, c = Statistical significant at *P < 0.05 *as compare to CCl_4_+ natansnin (10 mg).

The effect of CCl_4 _with and without the prior administration of natansnin on levels of catalase activity was shown in Figure 3. The activity of catalase decreased in both homogenate and mitochondria by 56 and 53% respectively in CCL_4 _treated rats. (Control = 733.9 ± 38.9 nmol/min/mg protein in homogenate and 1453 ± 64.6 nmol/min/mg protein in mitochondria. CCl_4 _= 323.7 ± 24.7 nmol/min/mg protein in homogenate and 685.05 ± 35.7 nmol/min/mg protein in mitochondria). But there is a significant increase in the catalase activity in both homogenate and mitochondria in rats administered with natansnin and CCl_4 _when compared to rats administered with only CCl_4_. Prior administration of natansnin at 10 mg/kg body wt protected them up to 35% (582.8 ± 30.1 nmol/min/mg protein) and 32% (1150.9 ± 54.8 nmol/min/mg protein) and at 20 mg/kg body wt the protection was slightly better 38.64% (593.6 ± 34.3 nmol/min/mg protein) and 36.06% (1190.8 ± 56.2 nmol/min/mg protein) in homogenate and mitochondria respectively. There was no significant effect on catalase levels in rats administered with natansnin alone. The effect of CCl_4 _in the presence and absence of natansnin on the activity of superoxide dismutase is shown in Figure 4. The activity of superoxide dismutase decreased in homogenate, mitochondrial fragment and mitochondria by 47 (1.51 ± 0.11 units/mg protein), 52 (1.48 ± 0.10 units/mg protein) and 57 (2.10 ± 0.25 units/mg protein) % respectively due to the effect of CCl_4_, when compared to normal rats (received only mineral oil). There was a considerable increase in the activity of SOD in all the fragments in rats fed with both concentrations of natansnin and CCl_4_, when compared to rats administered with only CCl_4_. The treatment of natansnin at lower dose (10 mg/kg body wt) protected the animals by 25% (2.22 ± 0.13 units/mg protein), 32% (2.47 ± 0.16 units/mg protein) and 32% (3.60 ± 0.19 units/mg protein) and at higher dose (20 mg/kg body wt) by 29% (2.30 ± 0.14 units/mg protein), 37% (2.47 ± 0.15 units/mg protein) and 36% (3.79 ± 0.23 units/mg protein) respectively. The activity of superoxide dismutase increased in natansnin treated rats, when compared to rats that were challenged with CCl_4. _Administration of natansnin alone did not show any change on SOD levels when compared to control animals.

### Natansnin restored cellular degeneration induced by CCl_4_

**Figure 5:**
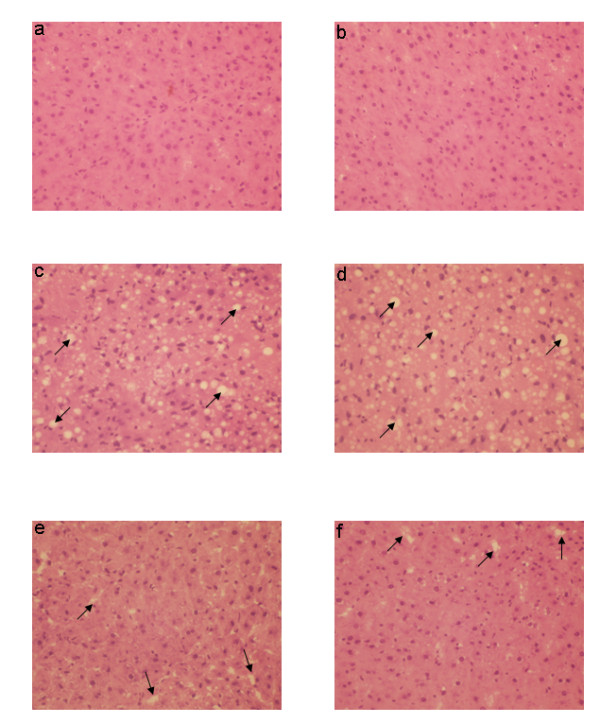
**Effect of CCl_4 _with or without prior administration of natansnin on histological characteristics**. Haematoxylin and Eosin staining was performed in liver sections of control (a, b), CCl_4 _(c, d) and natansnin (10 mg/kg (e), 20 mg/kg (f) body weight) treated rats. An Arrow symbol represents fatty vacuoles and lattice nature of the hepatocytes.

**Figure 6:**
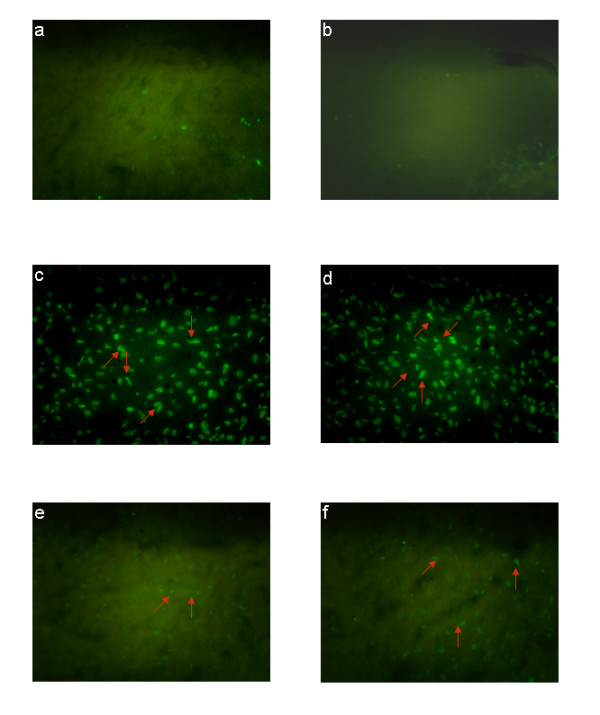
**Effect of CCl_4 _with or without prior administration of natansnin on cell death**. TUNEL analysis was performed for the location of the apoptotic cells in liver sections of control (a, b), CCl_4 _(c, d) and natansnin (10 mg/kg (e), 20 mg/kg (f) body weight) treated rats. TUNEL positive cells (apoptotic cells) indicated by arrows.

Paraffin wax sectioning and Haematoxylin-Eosin staining were performed for histopathological studies of rats. Haematoxylin-Eosin staining of control liver had normal cell morphology and is shown in Figure 5a and 5b. The histoarchitecture of hepatic cells of CCl_4 _challenged rats is shown in Figure 5c and 5d. The hepatic cells of natansnin and CCl_4 _treated rats are shown in Figure 5e and 5f. When compared to the histoarchitecture of the hepatocytes of control animals, hepatocytes of CCl_4 _challenged rats revealed extensive damage, characterized by the disruption of lattice nature of the hepatocyte, damaged cell membrane, degenerated nuclei and increased fatty vacuolation. In Group 4 and 5 rats (exposed to natansnin + CCl_4_), only minimal disruption of the hepatic cellular structure and less vacuolation were noticed. Prior administration of natansnin decreased the cellular degeneration, compared to CCl_4 _treated hepatocytes. Hepatocyte apoptosis in fixed liver specimens were analyzed by terminal dUTP nick- end labeling (TUNEL) assay. TUNEL sections of control, CCl_4 _treated and natansnin and CCl_4 _treated rats are shown in Figures 6a, b, c, d, e and 6f respectively. The number of TUNEL positive hepatocytes is more in CCl_4 _treated rats, when compared to controls indicating an increase in apoptotic degeneration of hepatocytes. Apoptosis in CCl_4 _treated liver was identified by the BrdU- FITC (green fluorescence at DNA nicks). BrdU-FITC is incorporated into the DNA breaks that were increased during free radical generation and apoptotic cell death pathway. Elevated levels of FITC fluorescent hepatocytes indicate the increased apoptosis of CCl_4 _treated liver hepatocytes over the control. The number of apoptotic cells was significantly reduced in both the concentrations of natansnin and CCl_4 _treated rats (6e and 6f) as evident from the decrease in TUNEL positive hepatocytes. Prior administration of natansnin decreased the number of apoptotic cells, when compared to CCl_4 _challenged hepatocytes (6c and 6d).

### Natansnin reduced the levels of apoptotic and inflammatory proteins

**Figure 7:**
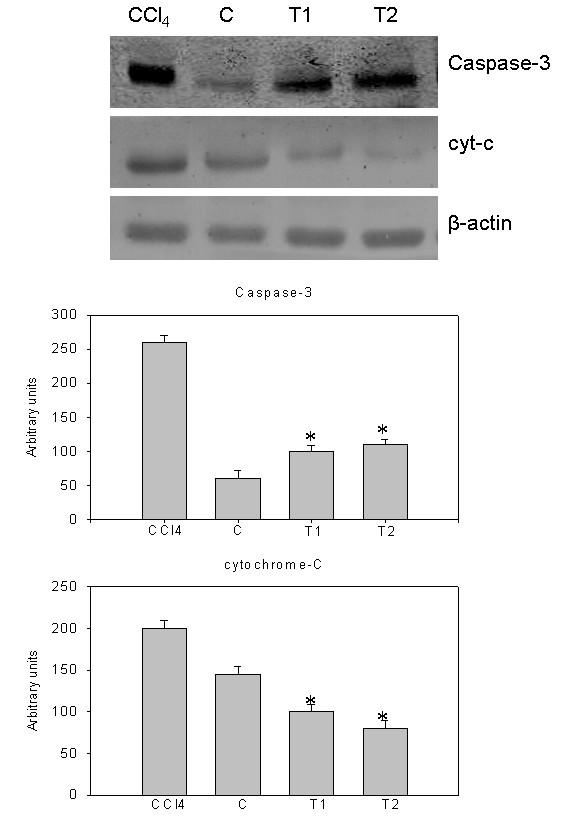
**Effect of CCl_4 _with or without prior administration of natansnin on Caspase-3 and cytochrome-C**. Whole cell protein extracted from control, CCl_4 _and natansnin (10 mg/kg, 20 mg/kg body weight) treated rats and separated on 12% SDS gels and transferred on to nitrocellulose membranes. Immunoblots were detected with Caspase-3 and cytochrome-C specific primary antibodies. The expression levels of β-actin were used as loading controls. Densitometric analysis showing the protein levels of Caspase-3 and cytochrome-C were increased in CCl_4 _group while treatment with natansnin significantly decreased the protein levels. Data are represented as mean ± standard deviation from three independent experiments (*p < 0.05 indicate significant difference relative to the CCl_4 _treated rats).

**Figure 8:**
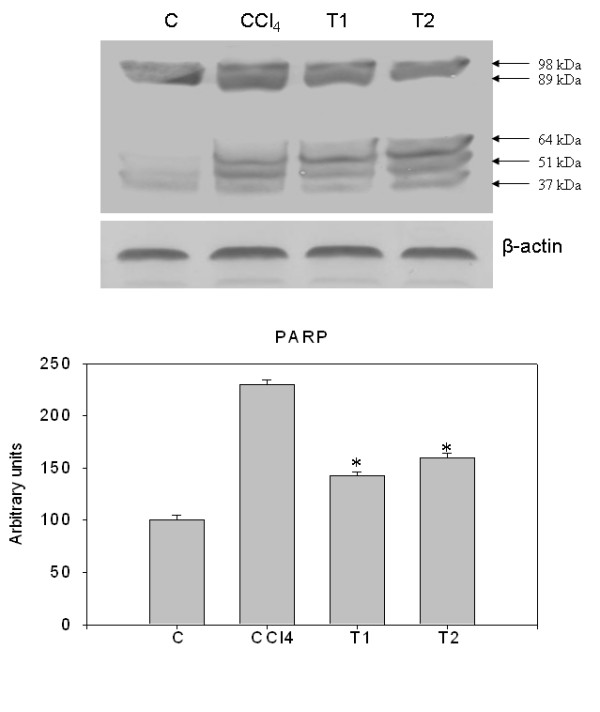
**Effect of CCl_4 _with or without prior administration of natansnin on PARP cleavage**. Whole cell protein extracted from control, CCl_4 _and natansnin (10 mg/kg, 20 mg/kg body weight) treated rats and separated on 12% SDS gels and transferred on to nitrocellulose membranes. Immunoblot was detected with PARP specific primary antibody. The expression levels of β-actin were used as loading controls. Densitometric analysis showing the protein levels of PARP were increased in CCl_4 _group while treatment with natansnin significantly decreased the protein levels. Data are represented as mean ± standard deviation from three independent experiments (*p < 0.05 indicate significant difference relative to the CCl_4 _treated rats).

**Figure 9:**
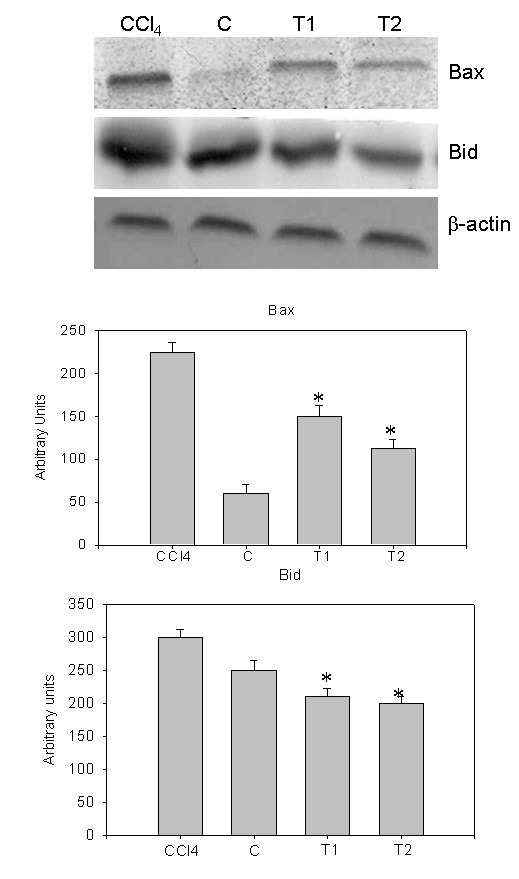
**Effect of CCl_4 _with or without prior administration of natansnin on Bax and Bid**. Mitochondrial protein extracted from control, CCl_4 _and natansnin (10 mg/kg, 20 mg/kg body weight) treated rats and separated on 10% SDS gels and transferred on to nitrocellulose membranes. Immunoblots were detected with Bax and Bid specific primary antibodies. The expression levels of β-actin were used as loading controls. Densitometric analysis showing the protein levels of Bax and Bid were increased in CCl_4 _group while treatment with natansnin significantly decreased the protein levels. Data are represented as mean ± standard deviation from three independent experiments (*p < 0.05 indicate significant difference relative to the CCl_4 _treated rats).

**Figure 10:**
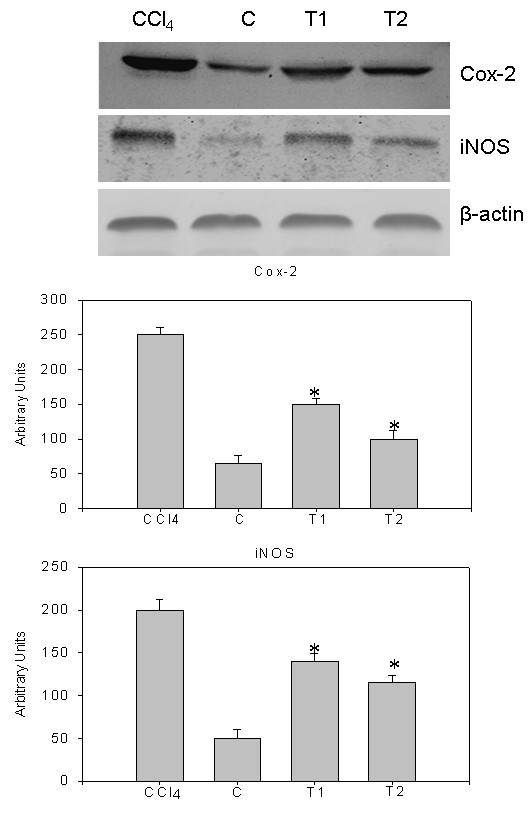
**Effect of CCl_4 _with or without prior administration of natansnin on COX-2 and iNOS**. Whole cell protein extracted from control, CCl_4 _and natansnin (10 mg/kg, 20 mg/kg body weight) treated rats and separated on 10% SDS gels and transferred on to nitrocellulose membranes. Immunoblots were detected with COX-2 and iNOS specific primary antibodies. The expression levels of β-actin were used as loading controls. Densitometric analysis showing the protein levels of COX-2 and iNOS were increased in CCl_4 _group while treatment with natansnin significantly decreased the protein levels. Data are represented as mean ± standard deviation from three independent experiments (*p < 0.05 indicate significant difference relative to the CCl_4 _treated rats).

Immunoblot analysis of caspase-3, PARP, cytochrome C, iNOS and COX-2 were performed in whole cell lysates of liver samples of all the animals. Also, immunoblot analysis of Bax and Bid were performed in mitochondrial fractions of all liver samples. Mitochondrial cytochrome C, detected as a single band of molecular mass (14 kDa) was increased in the whole cell lysate of CCl_4 _treated rats, compared to controls (Figure 7). Densitometric analysis of this protein is shown in Figure 7. Release of cytochrome C from mitochondria to cytosol is clearly noticed. Immuno blot analysis of pro caspase-3 (17 kDa) and active caspase-3 (32 kDa) increased in CCl_4 _treated rats and densitometric analysis of this protein is also shown in Figure 7. Immuno blot analysis of cleaved PARP fragments (37 kDa, 51 kDa, 64 kDa, 89 kDa and 98 kDa) increased in CCl_4 _treated rats and also its densitometric analysis is shown in Figure 8. The cleavage of PARP was clearly observed. The levels of these proteins were appreciably reduced in natansnin treated, compared to CCl_4 _exposed animals indicating a decrease in the cell death-signaling pathway. Immuno blot analysis of apoptotic proteins like Bax (21kDa) and Bid (23kDa) in mitochondrial fractions of CCl_4 _treated rats revealed an increase and are shown in Figure 9. The immune blot analysis of inflammatory proteins iNOS (130 kDa) and COX-2 (74 kDa) considerably increased in CCl_4 _treated rats and are shown in Figure 10. The densitometric analyses of these proteins are also shown in Figure 10. Natansnin treatment reduced the level of these inflammatory proteins. However, levels of these proteins reduced significantly in natansnin (both concentrations) treated rats, compared to CCl_4 _exposed ones indicating a decrease in the cell death.

## Discussion

CCl_4 _induced liver injuries are the best characterized system of xenobiotic-induced hepatotoxicity and for the screening of anti-hepatotoxic or hepatoprotective activities of drugs [16]. To our knowledge, this is the first study to demonstrate that prior administration of natansnin ameliorated CCl_4_-induced acute liver injury in rats as evidenced by both oxidative, cellular degeneration and cell death parameters mentioned above. Indeed, several natural products were reported with their protective efficacy against CCl_4_-induced hepatotoxicity [17]. The medicinal herbs *Artemisia campestris *[18] and *Phyllanthus fraternus *[19] have been found to scavenge free radicals and therein to exert a hepatoprotective effect against CCl_4_-induced liver injury. Free radicals, from both endogenous and exogenous sources, are implicated in the etiology of several degenerative diseases such as coronary artery disease, stroke, rheumatoid arthritis, diabetes and cancer [20].

The basis of CCl_4 _hepatotoxicity lies in its biotransformation by the cytochrome P-450 system to two free radicals. The first metabolite, a trichloromethyl free radical (.CCl_3_) [21] has been formed from the metabolic conversion of CCl_4 _and reacts very rapidly with O_2 _and forms a second metabolite, a trichloromethyl peroxy free radical (CCl_3_OO) [22] or abstract hydrogen atoms to form chloroform. These free radicals initiate the peroxidation of membrane poly-unsaturated fatty acids and covalently bind to microsomal lipids and proteins [23]. This phenomenon results in the generation of ROS like the superoxide anion O_2_^-^, H_2_O_2 _and the hydroxyl radical, .OH. ROS affect the antioxidant defense mechanisms, decrease the intracellular concentration of reduced glutathione (GSH) and reduces the activity of SOD and CAT.

In the present study, it was observed that intoxication of male Wister rats with CCl_4 _caused a marked rise in the level of serum enzymes like ALT and ALP, lipid peroxides and oxidized glutathione and caused significant decrease in GSH, SOD and CAT activities in liver. Prior treatment with natansnin displayed a profound effect. Lipid peroxidation leading to cell membrane damage is known to occur in CCl_4 _induced hepatotoxicity [24]. Membrane lipid peroxidation is an important pathophysiological event in a variety of diseases and stress conditions. MDA is a major reactive aldehyde produced from the peroxidation of polyunsaturated fatty acid present in the biological membranes [25]. It was hypothesized that CCl_4_- induced hepatotoxicity is mainly due to the lipid peroxidation of hepatocyte membranes by free radical derivatives of CCl_4 _[26]. The observation of elevated levels of hepatic MDA in Group 3 rats (administered with CCl_4 _alone) in the present study is consistent with this hypothesis. Natansnin at both concentrations brought down the elevated lipid peroxides which is of great value and provide an additional support to suggest the hepato-protective role of natansnin.

Mitochondrial GSH depletion may compromise mitochondrial function and sensitizes cells to diverse oxidant-induced toxicity, leading to cell death [27]. Liver injury included by consuming alcohol, by taking drugs like acetaminophen [28] or toxic chemicals like CCl_4 _are all known to be correlated with low tissue levels of GSH. In our study we observed a fall in the levels of reduced glutathione (GSH) and a rise in levels of oxidized glutathione (GSSG) in CCl_4 _challenged rats which are consistent with the results of other workers. Prior treatment of natansnin at both concentrations increased the levels of GSH and decreased the levels of GSSG when compared to CCl_4 _treated rats and these are close to the values obtained in controls. The ratio of GSH/GSSG is significantly restored in natansnin treated rats. Natansnin displayed hepatoprotection in natansnin treated rats with its antioxidant activity. In rats receiving CCl_4 _and natansnin, the activities of CAT and SOD were significantly higher than in CCl_4 _challenged rats, and are very similar to the values noted in normal rats. This suggests a hepatoprotective efficacy of natansnin, which is an interesting finding. The compound possibly confers this protective effect by diminishing the generation of free radicals induced by CCl_4_.

Histopathological studies were performed to provide direct evidence of the hepatotoxicity of CCl_4_. Marked disruption of the structure of hepatocytes was noted in liver tissue of Group II rats (exposed to CCl_4 _alone). Stripp et al., [29] reported that lipids were accumulated abnormally in the liver of CCl_4 _induced hepatotoxic rats. The hepatoprotective effect of natansnin was supported by histological examination. Only minimal disruption of the structure of hepatocytes was noted in liver tissue of rats exposed to CCl_4 _and natansnin. Elevated levels of FITC fluorescent hepatocytes indicate the increased apoptosis of CCl_4 _treated liver hepatocytes over the control. The number of apoptotic cells were significantly reduced in both the concentrations of natansnin treated rats as evident by the decrease in TUNEL positive hepatocytes. Liver has selective vulnerability to different toxins; hence we wanted to determine the mechanism of cell death in liver after CCl_4 _treatment. Results of the current study revealed increased caspase- 3 activation and PARP cleavage in CCl_4 _treated rats in comparison with controls. This indicates that the cell death mechanism involves caspase- 3 activation. In previous studies, loss of mitochondrial cytochrome C was correlated with an increased production of reactive oxygen species by mitochondria, which may contribute to cellular damage [30]. Prior treatment of natansnin profoundly decreased the increased expression of caspase- 3, PARP cleavage and translocation of cytochrome C from mitochondria to cytosol indicating that natansnin decreases the apoptosis in hepatocytes. Increased activation of Bax and Bid in CCl_4 _treated rats indicates apoptosis is more in these rats. Prior treatment of natansnin (both concentrations) reduced the incidence of apoptosis by decreasing the activation of Bax and Bid. Recent reports also demonstrated that induced nitric oxide overproduction occurs in the liver of rats with CCl_4_-induced acute liver injury suggested that iNOS may act as a mediator in the pathogenesis of hepatotoxicity in rats [31]. Increased expression of COX-2, a known inflammatory mediator and cancer has been observed in the present study. Increased expression of COX-2 and iNOS indicate that there is a rise in inflammation in CCl_4 _treated rats. Prior treatment of natansnin significantly reduced this inflammatory effect. The present study revealed that prior administration of natansnin (both concentrations) significantly decreased the CCl_4 _induced oxidative stress and cellular degeneration.

## Conclusions

The present study demonstrated that CCl_4 _induced a marked rise in oxidative stress and cellular degeneration in rat liver. Both the doses of natansnin treatment (10 and 20 mg/kg body wt) significantly ameliorated the effect of CCl_4 _induced oxidative stress damage and inhibited the expression of inflammatory and apoptotic proteins. This protective effect of natansnin can be correlated directly to its antioxidant property.

## Methods

### Chemicals and reagents

Thiobarbituric acid (TBA), epinephrine, GSH, GSSG, o-phthalaldehyde (OPT), N-ethyl maleimide (NEM), xanthine oxidase, PMSF, leupeptin, aprotinin, sodium orthovandate (Na_3_Vo_4_), Sodium fluoride (NaF), dithiotrietol (DTT), Na deoxycholate, β-glycerophosphate were obtained from Sigma chemical company, USA. Caspase-3, iNOS, COX-2, PARP, cytochrome C, Bax, Bid primary antibodies were obtained from Cell Signaling Technology, Beverly, MA, USA. Goat anti-rabbit and anti-mouse IgG ALP conjugate secondary antibodies, BCIP-NBT substrates for alkaline phosphatase, protein molecular weight marker were obtained from Genei Pvt Ltd, Bangalore, India. All other chemicals were obtained from standard commercial sources in India and were of analytical grade.

### Isolation of compounds from *Salvinia natans*

Solvents such as hexane, ethyl acetate, chloroform and methanol were used for extraction of the compounds. The method adopted to isolate the compounds from *Salvinia natans *was column chromatography. The plant material was shade dried for ten days and powdered as mentioned earlier. Approximately 5 kg of the dried plant powder was immersed in hexane first to defatt and then it was extracted in methanol. The methanol extract was concentrated by distillation on a water bath. The concentrate was dried for a day and little amount of chloroform was added till it became wet. Then silica of 60:120 mesh sizes was added to the wet concentrate. The compounds were adsorbed to silica. The sticky concentrate became powdery and it was dried at room temperature for 1 day to remove any solvent present in it. It was then loaded into a glass column of 3 feet length and 5 cm width filled with silica (100:200). The compounds were isolated from low polar to high polar compounds. All the fractions were monitored by recording ^1^H-NMR spectrum and the fractions contain glycoside signals were pooled and were further purified on silica gel followed by reversed phase C18 HPLC column to afford a novel dibenzoyl glycoside natansnin.

### DPPH radical method

A modification of the method of [32] was used. Ethanolic solutions of DPPH^- ^(10^-4 ^M) and natansnin or butylated hydroxy tolune (BHT) solutions were mixed in disposable plastic half-microcuvettes, so that the final mass ratios were natansnin: DPPH^- ^= 5.5:1, and reference compound (BHT): DPPH^- ^= 0.5:1. The samples were incubated for 15 min in the dark at 30°C and the decrease in absorbance at 517 nm was measured against ethanol using a spectrophotometer. All determinations were performed in triplicate. The radical scavenging activities of the tested samples, expressed as percentage inhibition of DPPH^-^, were calculated according to the formula percentage inhibition = [(AB _ AA)/AB]*100 [33] where AB and AA are the absorbance values of the blank and of the test sample, respectively, after 15 min.

### Treatment of experimental animals

Research on animals was carried out with approval from the Institutional Animal Ethics Committee (IEAC) guidelines. Male albino Wistar rats weighing 100 ± 20 g were taken from the animal house facility of University of Hyderabad and checked for proper growth for at least 8-10 days. They were fed with commercial pellet diet and tap water ad libido. The animals were divided into five groups of four rats in each group.

Group 1: Control rats, which received 0.5 ml of mineral oil orally for 8 days.

Group 2: Received CCl_4 _(4 g/kg body wt) in 0.5 ml of mineral oil intraperitoneally and sacrificed after 24 h.

Group 3: Received natansnin (20 mg/kg body wt) in 0.5 ml of mineral oil orally for a period of 8 days and sacrificed.

Group 4: Received natansnin (10 mg/kg body wt) in 0.5 ml of mineral oil orally for a period of 8 days and then CCl_4 _(4 g/kg body wt) was given and sacrificed after 24 h.

Group 5: Received natansnin (20 mg/kg body wt) in 0.5 ml of mineral oil orally for a period of 8 days and then CCl_4 _(4 g/kg body wt) was given and sacrificed after 24 h.

### Assessment of oxidative stress

#### Isolation of mitochondria

A slightly modified method of Laurence and Davies [34] was used for the preparation of mitochondria. Liver was homogenized followed by differential centrifugation in ice cold medium containing 220 mM D-mannitol, 70 mM sucrose, 2 mM HEPES, 0.2 mM EDTA and 0.36 mg/ml of bovine serum albumin and adjusted to pH 7.4. The final pellet containing mitochondria was suspended in 3 ml of 0.25 M sucrose and the protein content was determined by Lowry method [35]. Mitochondria were kept at -80°C until further use.

#### Determination of enzyme levels in serum

Alkaline phosphatase (ALP) activity (IU/L) in serum was measured using standard assay kits Radiant-Centronic GmbH (Germany) for ALP). Serum alanine aminotransferase (ALT) was estimated by International Federation of Clinical Chemistry [36] (ERBA test kits).

#### Lipid peroxidation measurement

In liver homogenate and mitochondria, lipid peroxide level was carried out by the method of Ohkawa et al [37]. A 10% liver homogenate was prepared in 1.15% Kcl and mitochondria were isolated by differential centrifugation. Mitochondria were washed with 1.15% Kcl and suspended in the same medium. Reaction system contained 5 mg of mitochondrial protein, 0.2 ml of 8.1% SDS, 1.5 ml of 20% acetic acid (pH 3.5) and 1.5 ml of 0.67% (w/v) aqueous solution of thiobarbituric acid. The total volume was made up to 4 ml with water and the tubes were heated in a waterbath at 95°C for 60 min. After cooling, 1.0 ml of water and 5.0 ml of n-butanol were added and the tubes were vortexed and then centifuged at 2000 × g for 10 min at room temperature. The absorbance of the organic layer was measured at 535 nm. A blank was also run simultaneously and tetra methoxy propane (TMP) was used as an external standard. The extent of lipid peroxidation was expressed as nmol of (MDA) formed per 100 mg of protein.

#### Determination of oxidized and reduced glutathione

Liver (250 mg) was homogenized in 3.75 ml of the phosphate - EDTA buffer (pH-8.0), 1 ml of 25% H_3_PO_4 _and centrifuged at 4°C at 100,000 g for 30 min and the supernatant obtained was used for the assay of reduced (GSH) and oxidised glutathione (GSSG) as per Hissin and Hilf [38].

#### Reduced glutathione (GSH) assay

To 0.5 ml of the supernatant, 4.5 ml of phosphate - EDTA buffer pH-8.0 was added. The final assay mixture contained 100 μl of the diluted tissue supernatant, 1.8 ml of phosphate - EDTA buffer (pH-8.0) and 100 μl of o-phthalaldehyde (OPT) solution. After 15 min incubation fluorescence at 420 nm was determined with the activation at 350 nm.

#### Oxidised glutathione (GSSG) assay

**T**he supernatant (0.5 ml) was incubated at room temperature with 200 μl of 0.04 M N-ethyl maleimide for 30 min. To this, 4.3 ml of 0.1N NaOH was added. To 100 μl of this mixture 1.8 ml 0.1 N NaOH, 100 μl of OPT was added and fluorescence was determined at 420 nm with activation at 350 nm. Results were expressed as μmoles per gm tissue.

#### GSH/GSSG Ratio

The GSH/GSSG ratio is calculated by dividing the difference between the GSH and GSSG concentrations by the concentration of GSSG.

Ratio=GSH−2(GSSG)/GSSG

#### Assay of catalase

Catalase activity was determined by the method of Aebi [39]. The assay mixture contained 150 μl of 30 mM H_2_O_2 _and 850 μl of enzyme source. The decrease in absorbance was measured immediately at 240 nm and activity was expressed as nmols of H_2_O_2 _consumed per min per mg protein.

#### Superoxide dismutase (SOD) determination

SOD activity was determined by the inhibition of oxidation of epinephrine to adrenochrome which was monitored at 480 nm using xanthine-xanthine oxidase system by the method of Dionisi et al [40]. Reaction mixture contained 0.01 M sodium carbonate buffer, 1 mM xanthine and 0.011 μM xanthine oxidase, 0.1-0.5 mg of protein sample (mitochondrial fragments or sonicated mitochondria or cytosol). The reaction was initiated by the addition of 1 mM epinephrine and absorbance was measured at 480 nm. Results are expressed as units per mg protein. One unit is defined as amount of enzyme which inhibits oxidation of epinephrine by 50%.

### Assessment of cellular degeneration

#### *In situ *cell death detection by TUNEL labeling

In situ detection of DNA fragmentation was done using the terminal deoxy nucleotidyl transferase-mediated dUTP-biotin nick end-labeling method (TUNEL) (BD Bio-sciences, San Jose, CA, USA) and the method was adopted as per the protocol given in the kit.

#### Western blotting

Rat liver tissues were homogenized in 5 volumes of radio immunoprecipitaiton assay (RIPA) buffer containing 50 mM Tris-HCl (pH 8.0), 150 mM NaCl, 1 mM EDTA, 0.4% deoxy cholate, 1% NP-40 containing protease inhibitors including 1 mM phenylmethylsulfonylfluoride (PMSF) and phosphatase inhibitors including 10 mM β-glycerophosphate, 10 mM NaF, 0.3 mM Na_3_Vo_4 _and 0.3 mM aprotinin. The lysate was sonicated for 2 min and centrifuged at 14,000 g for 15 min at 4°C. The supernatant was collected as whole tissue lysate and frozen at -80°C before use. Protein concentrations were determined by Lowry method. Cellular protein (75 μg) was mixed with SDS sample buffer, boiled for 5 min and subjected to electrophoresis on 10% SDS-polyacrylamide gels and transferred onto nitrocellulose membranes. After blocking the nitro cellulose paper in non-fat dry milk (5%) in Tris Buffered Saline, (TBS) 10 mM Tris (pH 7.5) and 150 mM NaCl for 1 h at room temperature, membranes were incubated for 12-24 h with Cox-2, iNos, Caspase-3, PARP, cytochrome C, Bax and Bid primary antibodies. Then membranes were incubated with secondary antibodies conjugated to alkaline phosphatase (ALP) (anti-rabbit and anti-mouse IgG conjugated to ALP obtained from Genei Pvt Ltd., Bangalore, India), for 1-2 h at room temperature. Before and after incubation with secondary antibodies, membranes were washed with TBS and TBST (TBS containing 0.1% Tween-20). Immunoreactivty was visualized by incubating the membranes with BCIP-NBT solution (Genei Pvt Limited, Bangalore, India). Membranes were analyzed quantitatively using image J software (NIH).

### Statistical analysis

All values were expressed as means ± S.D. Statistical significance was performed by analysis of variance followed by Bonferror's test. *P *< 0.05 was considered as significant.

## Abbreviations

MDA: malonaldehyde bis dimethylacetal; BSA: bovine serum albumin; EDTA: ethylene diamine tetraaceticacid; GSH: glutathione (reduced); GSSG: glutathione (oxidized); SOD: superoxide dismutase; PMSF: phenylmethylsulfonylfluoride; COX-2: cyclooxygenase-2; Cyt-C: cytochrome-C; ALP: alkaline phosphatase; ALT: alanine transaminase.

## Authors' contributions

PS: Responsible for the bench work

GRS: Responsible for the lab work

PBK: Provided facilities for some part of the work

OHS: Provided facilities for some part of the work

PPB: Provided complete lab facilities, financial assistance and necessary guidance

All authors read an approved the final draft.
